# Supplementary material for: Identification of photoperiod-induced specific miRNAs in the adrenal glands of Sunite sheep (Ovis aries)
Source: Front Vet Sci. 2022 Jul 22;9:888207. doi: 10.3389/fvets.2022.888207 (PMC9354845; doi:10.3389/fvets.2022.888207)
Supplement: Supplementary file 1 [file Data_Sheet_1.ZIP › Supplementary file/miRDeep2 file.zip!/miRDeep2 file/Know_Structure/SP42A2/oar-mir-432.pdf]

miRBase precursor : oar-mir-432
Total read count : 2220
oar-miR-432 read count : 2212
remaining reads : 8

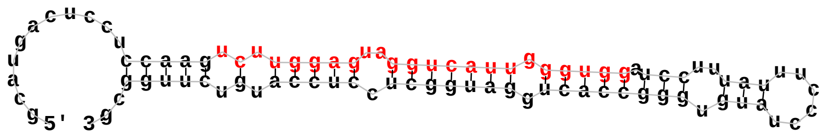

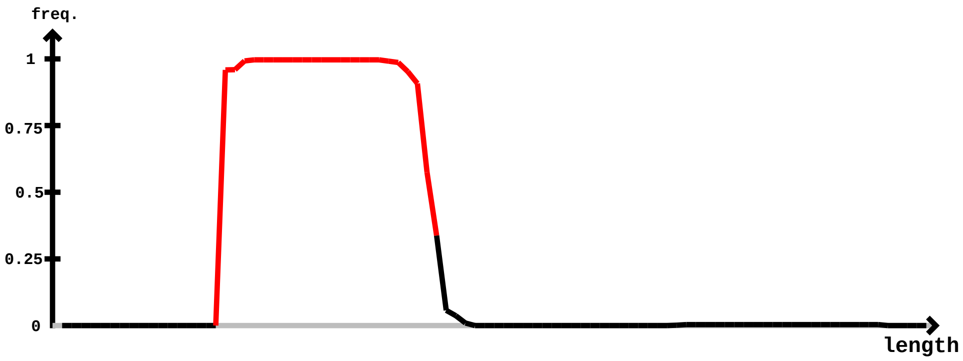


```
                 oar-miR-432
5'- gcaugacuccuccaagucuuggaguaggucauuggguggauccuuuauuucccuaugugggccacuggauggcuccuccaugucuuggcg   -3'    exp
    ..........(((((.(.(((((..(((((((.(((((.(((..(((......))).)))))))).))))))).))))).).)))))..    reads      mm         sample
    ................ucuuggaguaggucauu.........................................................    11         0          seq
    ................ucuuggaguaggucauug........................................................    11         0          seq
    ................ucuuggaguaggucauugg.......................................................    76         0          seq
    ................ucuuggaguaggucauuggg......................................................    99         0          seq
    ................ucuuggaguaggucauugggu.....................................................    737        0          seq
    ................ucuuggaguaggucauugggug....................................................    524        0          seq
    ................ucuuggaguaggucauuggguggg..................................................    618        0          seq
    ................ucuuggaguaggucauugggugga..................................................    38         0          seq
    ................ucuuggaguaggucauuggguggau.................................................    14         0          seq
    .................cuuggaguaggucauugggu.....................................................    1          0          seq
    .................cuuggaguaggucauuggguggg..................................................    1          0          seq
    ..................uuggaguaggucauugggug....................................................    3          0          seq
    ..................uuggaguaggucauuggguggg..................................................    3          0          seq
    ..................uuggaguaggucauugggugga..................................................    7          0          seq
    ..................uuggaguaggucauuggguggau.................................................    40         0          seq
    ..................uuggaguaggucauuggguggauc................................................    20         0          seq
    ...................uggaguaggucauugggug....................................................    1          0          seq
    ...................uggaguaggucauuggguggau.................................................    7          0          seq
    ...................uggaguaggucauuggguggauc................................................    1          0          seq
    ...............................................................acuggauggcuccuccaugucu.....    3          0          seq
    ................................................................cuggauggcuccuccaugucu.....    5          0          seq
```
